# Temporal Expression of Chemokines Dictates the Hepatic Inflammatory Infiltrate in a Murine Model of Schistosomiasis

**DOI:** 10.1371/journal.pntd.0000598

**Published:** 2010-02-09

**Authors:** Melissa L. Burke, Donald P. McManus, Grant A. Ramm, Mary Duke, Yuesheng Li, Malcolm K. Jones, Geoffrey N. Gobert

**Affiliations:** 1 Molecular Parasitology Laboratory, Queensland Institute of Medical Research, Herston, Queensland, Australia; 2 The School of Population Health, The University of Queensland, Herston, Queensland, Australia; 3 Hepatic Fibrosis Laboratory, Queensland Institute of Medical Research, Herston, Queensland, Australia; 4 Parasite Cell Biology Laboratory, Queensland Institute of Medical Research, Herston, Queensland, Australia; 5 The School of Veterinary Science, The University of Queensland, St Lucia, Queensland, Australia; Uniformed Services University, United States of America

## Abstract

Schistosomiasis continues to be an important cause of parasitic morbidity and mortality world-wide. Determining the molecular mechanisms regulating the development of granulomas and fibrosis will be essential for understanding how schistosome antigens interact with the host environment. We report here the first whole genome microarray analysis of the murine liver during the progression of *Schistosoma japonicum* egg-induced granuloma formation and hepatic fibrosis. Our results reveal a distinct temporal relationship between the expression of chemokine subsets and the recruitment of cells to the infected liver. Genes up-regulated earlier in the response included T- and B-cell chemoattractants, reflecting the early recruitment of these cells illustrated by flow cytometry. The later phases of the response corresponded with peak recruitment of eosinophils, neutrophils, macrophages and myofibroblasts/hepatic stellate cells (HSCs) and the expression of chemokines with activity for these cells including *CCL11* (eotaxin 1), members of the Monocyte-chemoattractant protein family (*CCL7*, *CCL8*, *CCL12*) and the Hepatic Stellate Cell/Fibrocyte chemoattractant *CXCL1*. Peak expression of macrophage chemoattractants (*CCL6*, *CXCL14*) and markers of alternatively activated macrophages (e.g. *Retnla*) during this later phase provides further evidence of a role for these cells in schistosome-induced pathology. Additionally, we demonstrate that CCL7 immunolocalises to the fibrotic zone of granulomas. Furthermore, striking up-regulation of neutrophil markers and the localisation of neutrophils and the neutrophil chemokine S100A8 to fibrotic areas suggest the involvement of neutrophils in *S. japonicum*-induced hepatic fibrosis. These results further our understanding of the immunopathogenic and, especially, chemokine signalling pathways that regulate the development of *S. japonicum*-induced granulomas and fibrosis and may provide correlative insight into the pathogenesis of other chronic inflammatory diseases of the liver where fibrosis is a common feature.

## Author Summary

Schistosomiasis, a disease caused by parasitic worms, is a significant cause of illness and death in the developing world. Furthermore, recent reports suggest that the global burden of disease due to schistosomiasis has been significantly underestimated. Schistosomiasis of the liver arises due to inflammation and the deposition of scar tissue around parasite eggs trapped in this organ. In the current study we analysed the gene-expression profile of the mouse liver at several time points following infection with a virulent strain of *Schistosoma japonicum* to better understand the mechanisms that regulate this process. Progression of disease was associated with increased expression of different groups of genes with distinct biological functions. Specifically, we identified several genes encoding chemical signalling molecules that contribute to different phases of the response by recruiting key cell types to the site of inflammation. This study represents the most comprehensive report to date of the gene expression profile in the liver during schistosomiasis. These results provide further insight into the mechanisms that regulate the development of schistosome-induced inflammation and scarring and will aid in the development of novel treatments to alleviate the burden of disease caused by this parasite.

## Introduction

Schistosomiasis, a parasitic disease caused by trematodes of the genus *Schistosoma*, is a significant cause of human morbidity and mortality. Furthermore, recent reports suggest that the global burden of disease due to schistosomiasis has been significantly underestimated [1]. Chronic infection with *S. mansoni* or *S. japonicum* leads to hepatosplenic schistosomiasis, periportal fibrosis, portal hypertension, hepatosplenomegaly, ascites and gastrointestinal bleeding that may lead to death [2]. Murine models of *S. mansoni* infection indicate that most pathology is attributable to a CD4+ Th2 driven granulomatous response against schistosome eggs and the antigens they secrete (reviewed in [2]). Early studies suggested that the basic immunopathogenic mechanisms associated with granuloma development and fibrosis were similar in both *S. mansoni* and *S. japonicum* infections (e.g. [3]), although the severity and features of infection with these two parasites are known to differ in a number of ways. *S. japonicum* eggs cluster in the host liver where they induce a more severe granulomatous response that is more neutrophilic than those induced by *S. mansoni* [4]. Furthermore, the immune response of infected mice to purified secreted egg antigen (SEA) is of a delayed type hypersensitivity reaction with *S. mansoni* infection but is of an immediate type hypersensitivity reaction with *S. japonicum* [5], suggesting there may be key differences in the pathways leading to granuloma formation and hepatic fibrosis caused by the two species.

We used microarray analysis of the mouse liver during infection with a highly pathogenic Chinese mainland field strain of *S. japonicum* to better define the molecular mechanisms involved in schistosome-induced immunopathology. Progression of disease from the onset of egg laying through to the development of mature granulomas and hepatic fibrosis was associated with temporal expression of genes with distinct biological functions. The contribution of different chemokine subsets to the pathogenesis of schistosomiasis was further defined and suggests that hepatic stellate cell, macrophage and neutrophil chemotaxis are important in the pathogenesis of schistosome-induced hepatic fibrosis.

## Materials and Methods

### Ethics Statement

All work was conducted with the approval of the Queensland Institute of Medical Research Animal Ethics Committee.

### Mice and Parasites

Four to six week old female C57BL/6 mice were percutaneously infected with 20 *S. japonicum* cercariae (Chinese mainland strain, Anhui population). Mice were euthanized at 4 (n = 7), 6 (n = 7) and 7 (n = 8) weeks post infection (p.i) and their livers perfused to obtain adult worms. Three additional mice were used as uninfected controls. An identical time-course experiment was performed for flow cytometry (n = 5 per group). The number of adult worm pairs per mouse was recorded as a measure of parasite burden. Eggs per gram of liver were calculated as a measure of hepatic egg burden as described [6]. Briefly, eggs were extracted from a portion of liver of known mass by overnight digestion in potassium hydroxide. Eggs were then resuspended in 1mL of formalin and the number of eggs in three 5µL aliquots were counted and averaged to calculate eggs per gram of liver.

### Histological Assessment

Formalin fixed, paraffin embedded liver sections were stained with Haematoxylin and Eosin (H&E), picosirius red for collagen as a measure of fibrosis, α-smooth muscle actin (SMA) immunoperoxidase staining for myofibroblasts/Hepatic Stellate Cells (HSCs) [7], Giemsa for eosinophils and Leder stain for neutrophils [8]. Slides were digitised using the Aperio Slide Scanner (Aperio Technologies, Vista, USA). Granuloma volume density, percent collagen staining (degree of fibrosis) and percent positive α-SMA staining were quantified by point counting stereology on H&E, picosirius red, and α-SMA stained slides respectively, where myofibroblasts/HSCs were defined as α-SMA positive, spindle shaped cells associated with focal areas of inflammation [7]. Semi-quantitation of eosinophils and neutrophils was performed by determining the mean number of positive-stained cells over 20 fields at high magnification (cells/hpf) (×400).

### Immunohistochemistry for Selected Chemokines

Immunohistochemistry for S100A8 and CCL7 was performed on paraffin embedded sections using commercially available primary antibodies (Santa Cruz Biotechnology Inc, Santa Cruz, USA) and detection kits (Biocare Medical, Concord, USA). Positive staining was quantified using Aperio's Spectrum Plus software positive pixel count algorithm (Version 8.2.395.1255; Aperio Technologies, Vista, USA).

### RNA Extraction and Pooling for Microarray Analysis and Real-time PCR

Each mouse group was normalised for egg burden by log transformation with outliers excluded on the basis of 95% confidence intervals. Total RNA was extracted from liver tissues using Trizol (Invitrogen, Carlsbad, USA) and an RNeasy Mini Kit (Qiagen Inc, Valencia, USA) [9]. Total RNA quantity was measured using a Nanodrop-1000 (Nanodrop Technologies, Wilmington, USA) and quality was assessed using an Agilent Bioanalyzer (Agilent Technologies, Foster City, USA). Equal amounts of four total RNA samples of the highest quality from each group were pooled for cRNA and cDNA synthesis.

### Microarray Analysis

#### cRNA synthesis and whole genome microarray analysis

cRNA was synthesised using the Illumina Total Prep RNA Amplification kit (Ambion Inc., Austin, USA). Microarray analysis was performed using Illumina Mouse 6 version 1.1 Whole Genome Expression Chips (Illumina, San Diego, USA). Two technical replicates were performed for each cRNA sample. All gene expression data are publicly available (NCBI's Gene Expression Omnibus; Series Accession Number: GSE14367).

#### Data analysis

Quality control of microarray data involved examination of intensity histograms of hybridisation efficiency and noise using BeadStudio, version 3 (Illumina, San Diego, USA). All subsequent analyses were performed using GeneSpring GX, version 7.3.1 (Agilent Technologies, Foster City, USA). Expression values were normalised to the median and 50^th^ percentile. Values less than 0.01 were set to 0.01. The data were then normalised to uninfected controls and filtered for significant signal on the basis of detection score (d>0.949, which equates to a confidence value of p≤0.05). At least 4 of 8 hybridisations had to pass these filtering criteria for a gene to be accepted. Analysis of variance (ANOVA, p≤0.05 using Benjamini and Hochberg correction for multiple testing) identified genes whose expression changed significantly over time. Hierarchical clustering using Pearson correlation measure on ANOVA filtered data identified common patterns of temporal gene expression. Correlations between the expression patterns of specific genes were assessed using Spearman's correlation. Keyword based searches for ‘chemokine’, ‘chemotaxis’ or specific gene names within the gene ontology or gene description were used to identify chemokines and other genes of interest.

#### Ingenuity pathway analysis

Ingenuity Pathway Analysis, version 6 (Ingenuity Systems, www.ingenuity.com) was used to identify biological functions and metabolic or signalling pathways within the Ingenuity Pathways Knowledge Base that were over-represented by genes in each of the identified gene clusters (Fischer's exact test, p≤0.05). A cut off of ±2-fold change in expression was applied allowing identification of changes in gene expression with likely biological significance.

### cDNA Synthesis and Real-time PCR

cDNA was synthesised using a Quantitect Reverse Transcription kit (Qiagen Inc., USA). cDNA concentration was measured using a Nanodrop-1000 (Nanodrop Technologies, Wilmington, USA.).

Real time PCR was used to validate a subset of microarray data. Genes and primers used for real time PCR were representative of transcripts that were significantly up or down-regulated during microarray analysis and were sourced from the literature [10],[11],[12],[13],[14],[15] or designed using Primer 3 software (http://biotools.umassmed.edu/bioapps/primer3_www.cgi) (Table S1). Hypoxanthine phosphoribosyltranferase (HPRT) was used as a housekeeping gene. Real time PCR was performed using SYBR Green master mix (Applied Biosystems, Warrington, UK) on a Corbett Rotor Gene 6000 (Corbett Life Sciences, Concorde, Australia). Rotor-Gene 6000 Series software (version 1.7) and Microsoft Office Excel 2003 were used to analyse the results. Correlation between real-time PCR and microarray data was performed in GraphPad Prism Version 5.00 (GraphPad Software, San Diego, USA) using Spearman's Rho measure of correlation as described [16].

### Isolation of Leukocytes from Liver Tissue

Leukocytes were isolated from liver tissue as described [17]. Briefly, liver tissue was digested in collagenase D (Roche Diagnostics, Mannheim, Germany) (1mg/ml) and DNAse I (0.5mg/mL, Roche Diagnostics, Mannheim, Germany) for 45 mins at 37°C. The tissue was then passed through a 70µm cell strainer (BD Falcon, Bedford, USA) and washed with phosphate buffered saline supplemented with 2% (v/v) Foetal Calf Serum (FCS). The cell pellet was then resuspended in 33% Percoll (w/v) and centrifuged at 1700rpm at room temperature to remove hepatocytes and other debris. The resulting leukocyte pellet was then washed in 2% FCS and red blood cells were lysed with Gey's lysis solution. The solution was then underlayed with 2% (v/v) FCS and centrifuged at 1300rpm for 5 mins. The resulting cell pellet was resuspended in FACS buffer (1% bovine serum albumin (w/v), 0.1% sodium azide (v/v) in phosphate buffered saline) and the cells were counted.

### Flow Cytometry

Leukocytes were stained for specific cell markers by first incubating with antibodies against the Fc-receptorIII (Monoclonal antibody producing hybridoma; Clone: 24.G2) to block non-specific binding and then with commercially available fluorochrome-conjugated antibodies (APC-anti-F4/80, PE-Cy5-anti-CD11b: Biolegend; APC-anti-CD4, FITC-anti-CD8b, PE-anti-CD19: BD Pharminogen; FITC-CD3, Miltenyi Biotec, Germany). Cell populations were defined as CD4+ T-cells (CD4+CD3+), CD8+ T-cells (CD8+CD3+), B-cells (CD19+) and Macrophages (F4/80+CD11b+). Data were acquired on FACS Calibur Flow Cytometer (BD Bioscience) and analysed using FlowJo Software (Treestar Inc) and GraphPad Prism, version 5.0 (GraphPad Software, San Diego, USA).

### Statistical Analysis

Changes in parasitological, histological and real time PCR data were assessed by One-Way ANOVA with post hoc Bonferroni testing (p≤0.05). These analyses were performed using the GraphPad Prism Version 5.00 (GraphPad Software, San Diego, USA).

## Results

### Parasitological and Histological Analyses

**Figure 1:**
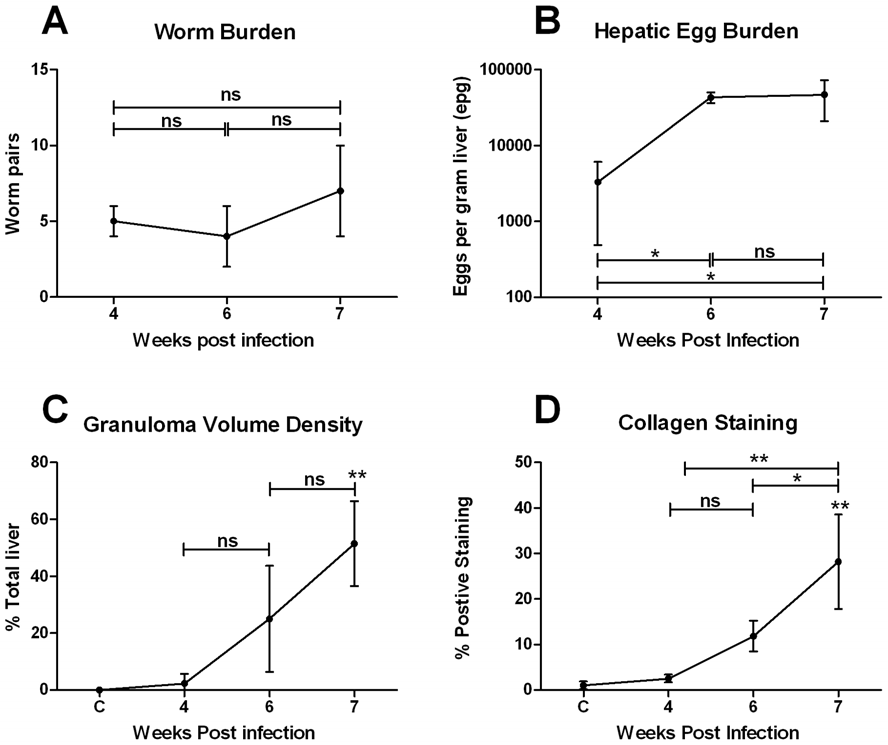
Parasite burden and granulofibrotic pathology. Infected mice harboured a mean of 5 worm pairs (A). Schistosome eggs were first observed in the liver at 4 weeks p.i and hepatic egg burden increased significantly thereafter (1-Way ANOVA, p≤0.05) (B). Granuloma volume (C) and collagen staining for hepatic fibrosis (D) increased significantly from 4 weeks p.i, reaching 51% and 28% total liver volume at 7 weeks p.i, respectively (1-Way ANOVA, p≤0.01). Values represent mean values from 4 mice pooled for microarray analysis ±1SD.* p≤0.05, ** p≤0.01, ns = not significant compared with uninfected liver unless otherwise indicated.

**Figure 2:**
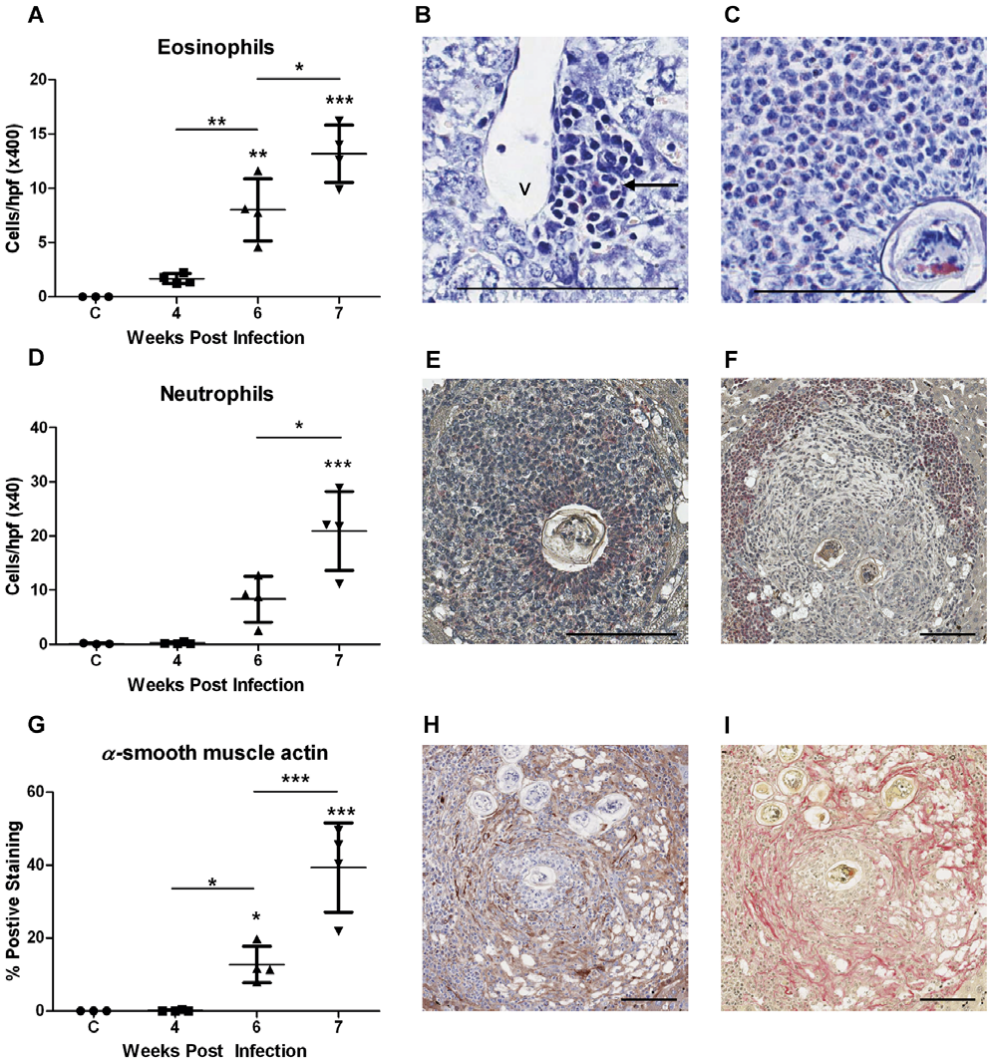
Changes in the distribution of eosinophils, neutrophils and myofibroblasts/HSCs in the murine liver during *S. japonicum* infection. The number of eosinophils in the liver increased significantly from 4 weeks p.i (A). Eosinophils (arrows) were first observed within small inflammatory infiltrates adjacent to blood vessels (V) (B: Giemsa ×400) and, later, within granulomas associated with schistosome eggs (C: Giemsa ×400). Neutrophils were first observed in the liver at 6 weeks p.i in small inflammatory foci and their numbers increased significantly thereafter (D). At 7 weeks p.i. neutrophils occurred in the centre of established granulomas adjacent to schistosome eggs (E: Leder stain ×200) and at the periphery of more fibrotic granulomas (F: Leder stain ×100). α-SMA staining for myofibroblasts/HSCs increased significantly in infected compared with uninfected liver at 7 weeks p.i (G) and was localised to the fibrotic zone of granulomas (H: α-SMA staining, ×100; I: Picosirius red staining of the same granuloma, ×100). Scale bar equals 100 µm. Values represent means of 4 samples pooled for microarray analysis ±1SD. *p≤0.05, **p≤0.01, ***p≤0.001 compared with uninfected liver unless otherwise indicated.

Infected mice harboured a mean of 5 worm pairs (Figure 1a). Schistosome eggs were first observed in the liver at 4 weeks p.i and hepatic egg burden increased significantly thereafter (1-Way ANOVA, p≤0.05) (Figure 1b). Granuloma volume increased significantly from 4 weeks p.i, reaching 51% total liver volume at 7 weeks p.i (1-Way ANOVA, p≤0.01) (Figure 1c). Hepatic fibrosis, as measured by collagen staining, in the livers of mice at 4 weeks p.i (3% total liver volume) was not significantly different to that of uninfected controls (1% total liver volume) (Figure 1d). Fibrosis increased from 4–6 weeks post infection but this change was not significant (6 weeks: 12% p>0.05 1 Way ANOVA). The degree of hepatic fibrosis increased further at 7 weeks p.i and was significantly greater than all other time points at 28% total liver volume (p≤0.05, 1-Way ANOVA). Eosinophil numbers increased significantly from 4 weeks p.i. (p≤0.01) (Figure 2a). Eosinophils were first observed in small inflammatory infiltrates adjacent to blood vessels and, later, within mature granulomas (Figure 2b,c). Numbers of neutrophils in the liver increased significantly from 6 weeks p.i. (Figure 2d). Neutrophils were first observed in small inflammatory infiltrates (6 weeks p.i.) and, later, in the centre of established granulomas adjacent to schistosome eggs and at the periphery of more fibrotic granulomas (7 weeks p.i.; Figure 2e,f). α-SMA staining for myofibroblasts/HSCs was localised to the fibrotic zone of granulomas and was increased significantly compared with uninfected controls at 7 weeks p.i (1-Way ANOVA, p≤0.001) corresponding with increased collagen staining and the development of fibrosis (Figure 2g–i).

### Microarray Analysis

#### Filtering of microarray data

Normalised data for each of the 46,643 genes on the Illumina microarray were filtered for significant signal and normalised to uninfected controls reducing the data set to 17,807 genes, of which, 4,692 were shown to be differentially expressed.

#### Hierarchical clustering

**Figure 3:**
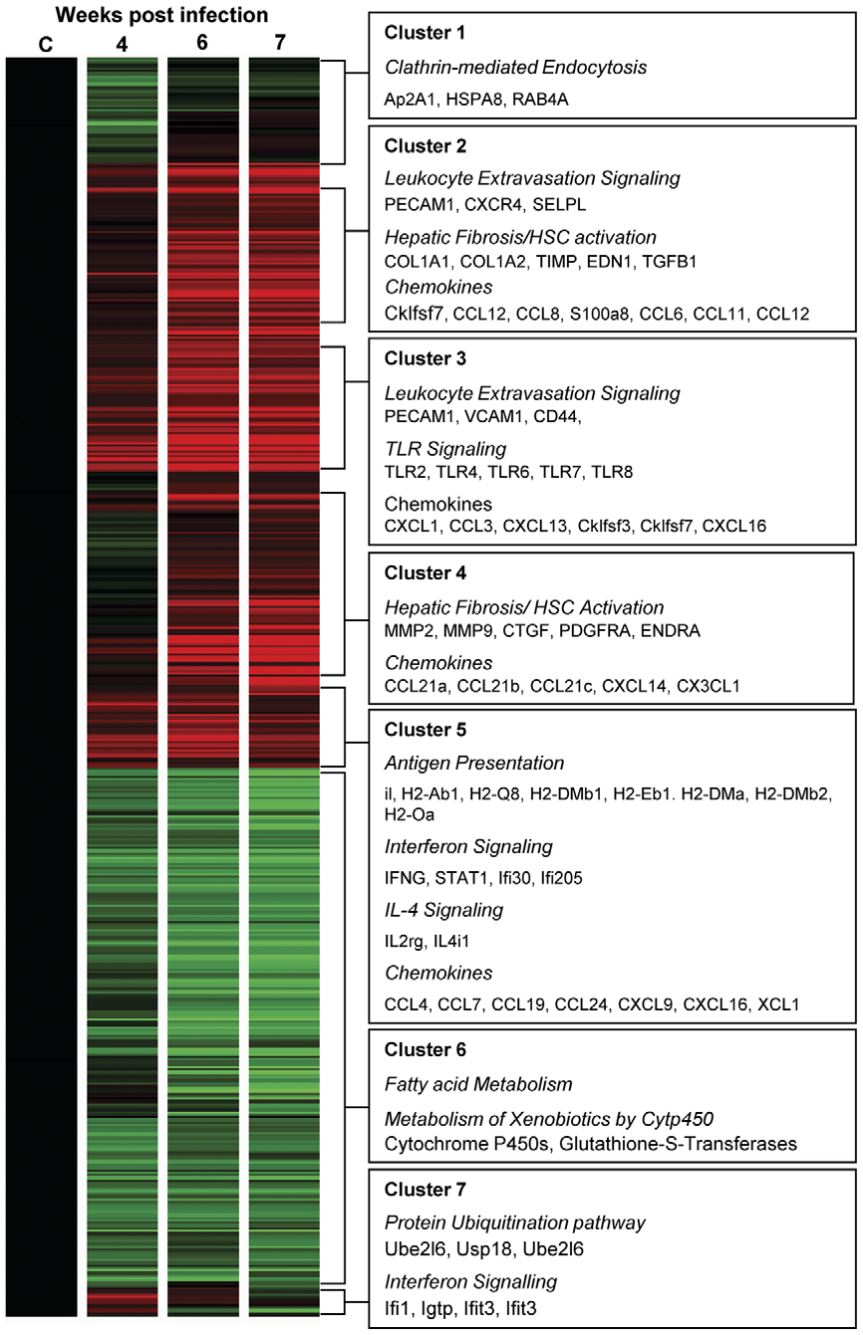
*S. japonicum* infection is associated with temporal expression of genes with distinct biological functions. Hierarchical clustering identified seven distinct clusters representing genes that were consistently up-regulated during infection (Cluster 3); up-regulated to a greater extent later in infection (Cluster 2 and Cluster 4); up-regulated to a greater extent earlier in infection (Cluster 5 and Cluster 7); and down-regulated during infection (Cluster 1 and Cluster 6). Prominent biological functions, signalling pathways or chemokines associated with each of these clusters are listed in the boxed text. Gene expression is represented as a heat map with relatively unchanged genes coloured black, down regulated genes coloured green and up-regulated genes coloured red.

Hierarchical clustering combined with Ingenuity Pathway analysis identified four distinct patterns of gene expression, represented by seven different clusters that were associated with distinct biological functions and signalling pathways (Figure 3 and Tables S2 and S3). Most striking was the temporal expression of different chemokine subsets (Table 1).

**Table 1:** Schistosomiasis is associated with temporal expression of different chemokine subsets.

	Expression+				
Chemokine Symbol	Illumina Probe ID	Accession #	4 weeks	6 weeks	7 weeks
***Up-regulated early***					
Cxcl9	105860594	NM_008599	16.4	6.7	2.4
Cxcl9	1570673	NM_008599	19.2	9.8	3.1
Xcl1	3800504	NM_008510	2.0	2.0	1.2
***Sustained Up-regulation***					
Ccl19	5080487	NM_011888	1.6	2.1	1.7
Ccl24	670129	NM_019577	6.5	10.0	6.3
Cklfsf3	1940180	NM_024217	2.2	3.8	3.7
***Peak expression 6 weeks p.i***					
Ccl3	2810092	NM_011337	1.8	11.3	4.7
Ccl4	50368	NM_013652	1.8	4.9	2.4
Ccl4	430047	NM_013652	4.4	20.6	8.3
Ccl7	2650519	NM_013654	2.1	5.4	2.8
Cxcl7	5130446	NM_023785	1.2	8.8	3.5
Cxcl13	6290402	NM_018866	3.1	20.1	7.6
***Up-regulated later***					
Ccl6	6660390	NM_009139	1.7	6.1	8.5
Ccl8	3870010	NM_021443	2.5	10.8	20.2
Ccl11	1770347	NM_011330	1.5	7.8	9.1
Ccl12	7000592	NM_011331	1.5	3.6	3.7
Ccl12	2120746	NM_011331	1.3	2.1	2.6
Ccl21a	5050338	NM_011335	1.2	1.4	4.2
Ccl21b	4670176	NM_011124	1.1	1.3	4.1
Ccl21c	6770037	NM_023052	1.2	1.3	3.9
Cxcl1	2690537	NM_008176	2.9	17.7	12.0
Cxcl13	6290402	NM_018866	3.1	20.1	7.6
Cxcl14	840114	NM_019568	0.9	3.3	7.1
Cxcl14	6450324	NM_019568	0.9	4.7	12.3
Cxcl16	510278	NM_023158	2.6	4.9	4.3
Cxcl16	100450458	NM_023158	1.8	2.3	2.0
Cx3cl1	3990707	NM_009142	1.1	1.5	2.4
Cklfsf7	4060114	NM_133978	2.0	4.6	6.0
Cklfsf7	5900358	NM_133978	2.4	6.0	7.1
S100a8	70112	NM_013650	3.3	90.8	141.8
S100a9	7050528	NM_009114	2.1	62.2	153.0
***Down-regulated***					
Ccl27	2120070	NM_011336	0.6	0.5	0.4
Cklfsf8	6200037	NM_027294	0.4	0.3	0.3
***Not significant or not detected***					
Ccl1	4230167	NM_011329	–	–	–
Ccl2	4760019	NM_011333	–	–	–
Ccl5*	3710397	NM_013653	3.3	3.4	1.5
Ccl9	4610725	NM_011338	–	–	–
Ccl17	630121	NM_011332	–	–	–
Ccl20	105130300	NM_016960	–	–	–
Ccl22*	6380086	NM_009137	1.2	1.1	1.4
Ccl25	540435	NM_009138	–	–	–
Ccl25*	450541	NM_009138	0.9	0.8	0.6
Ccl26l	380739	NM_001013412	–	–	–
Ccl28	2690593	NM_020279	–	–	–
Cklfsf2a	4920348	NM_027022	–	–	–
Cklfsf2b	1690041	NM_028524	–	–	–
Cklfsf4	360600	NM_153582	–	–	–
Cklfsf4*	3520332	NM_153582	0.9	1.0	0.9
Cklfsf6*	6590010	NM_026036	0.5	0.6	0.6
Cxcl2*	610398	NM_009140	1.3	5.9	1.9
Cxcl4*	6130332	NM_019932	0.6	3.8	1.2
Cxcl5	6370333	NM_009141	–	–	–
Cxcl10	2450408	NM_021274	–	–	–
Cxcl11	1090551	NM_019494	–	–	–
Cxcl12	580546	NM_021704	–	–	–
Cxcl12	4570068	NM_021704	–	–	–
Cxcl12	4150750	NM_021704	–	–	–
Cxcl15	2680451	NM_011339	–	–	–
Cxcl16*	101780301	NM_023158	1.1	1.4	1.2
Scyb11	103610324	AK050012	–	–	–

+Expression values were generated from microarray data and are displayed as a ratio relative to uninfected mice.

*No significant difference in expression over time by 1-Way ANOVA p>0.05.

– equals not detected.

#### Down-regulated genes are associated with metabolism

Genes down-regulated in response to *S. japonicum* infection (Clusters 1 and 6) included multiple components of many metabolic pathways including, but not restricted to, key components of detoxification pathways and the fatty acid and amino acid metabolic pathways.

#### Early gene expression is associated with both Th1 and Th2 responses

Expression of genes in cluster 5 peaked at 4 or 6 weeks p.i. corresponding with the initiation of the granulomatous response. Genes within this cluster, were associated with both Th1 (*IFN-γ* (NM_008337), *STAT 1* (NM_009283), *CXCL9*, *IFN-γ-induced GTPase* (*Igtp*; NM_01873)) and Th2 responses (IL4-induced 1 (*Il4i1*; NM_010215), *CCL24*, *CCL7*). Expression of Th1 associated genes peaked between 4–6 weeks p.i and declined thereafter, whereas expression of Th2 associated genes exhibited moderate peak in expression at 6 weeks p.i. A number of chemokine genes including *CXCL9*, *CCL4*, *CCL7*, *CCL19*, *CCL24* and *XCL1* also occurred in this cluster. Expression of *CXCL9*, *XCL1* correlated with expression of genes encoding components of the T-cell receptor complex (e.g. *CD3e* (NM_007648)) while *CCL19* expression correlated with those of B-cell markers including *CD22* (NM_009845). *CCL7* expression was correlated with that of alternatively activated macrophage markers and fibrosis associated genes (Table 2).

**Table 2:** Chemokine expression correlated with that of target cell markers and pro-fibrotic genes.

Gene of Interest	Chemokines	*r-value	*p-value
**Cell Markers**			
*Resistin-like-alpha (Retnla; NM_020509)*	*CCL6*	1.0000	<0.0001
	*CCL8*	0.9762	0.0004
	*CCL12*	0.9762	0.0004
	*CXCL14*	0.7857	0.0279
*Neutrophil Elastase (NE; NM_015779)*	*CXCL1*	0.7619	0.0368
	*S100A8*	0.9286	0.0022
	*S100A9*	0.9286	0.0022
*CD22 (NM_009845.1)*	*CXCL13*	0.9286	0.0022
	*CCL19*	0.8810	0.0072
*CD3e (NM_007648*)	*CXCL9*	1.0000	<0.0001
	*XCL1*	0.9048	0.0046
*Eosinophil Peroxidase (EPX; NM_007946)*	*CCL11*	0.7597	0.0287
**Fibrosis associated genes**			
*Procollagen, type-1-α-1 (COL1A1; NM_007742)*	*CXCL1*	0.7381	0.0458
	*CCL3*	0.7619	0.0368
	*CCL7*	0.7619	0.0368
*TGF-β (NM_011577)*	*CXCL1*	0.9762	0.0004
	*CCL3*	0.9524	0.0011
	*CCL7*	0.9524	0.0011
*PDGF-β (NM_011057)*	*CXCL1*	1.0000	<0.0001
	*CCL3*	0.9762	0.0004
	*CCL7*	0.9762	0.0004

*Spearman's correlation of gene expression data from microarray analysis, n = 8 all comparisons.

#### Genes consistently up-regulated are associated with immune responses

Genes demonstrating sustained up-regulation during the granulofibrotic response (Cluster 3) were significantly associated with immunological functions including leukocyte extravasation signalling, antigen presentation through the MHC class II complex, the acute phase response and Toll-like receptor signalling.

Genes for chemokines with activity for several cell types including B-cells (*CXCL13*), T-cells (*CXCL16*), neutrophils/Hepatic Stellate Cells (HSCs) (*CXCL1*) and leukocytes (*CCL3*) also grouped within Cluster 3. *CXCL13*, *CXCL16*, *CXCL1* and *CCL3* were significantly up-regulated at all time points (1-way ANOVA, p≤0.05) with a moderate, but significant, increase in expression at 6–7 weeks p.i (1-way ANOVA, p≤0.05) coinciding with the onset of fibrogenesis. *CXCL1* exhibited the greatest increase in expression reaching 18-fold at 6 weeks p.i. *CXCL1* and *CCL3* expression correlated with expression of several procollagen genes and the profibrotic cytokines *TGF-β1* (NM_011577) and *PDGF-β* (NM_011057). *CXCL13* expression correlated well with those of genes encoding B-cell markers, such as *CD22* (Table 2).

#### Late gene expression is associated with fibrosis

Genes in clusters 2 and 4 exhibited peak expression at 6–7 weeks p.i paralleling increased collagen deposition and hepatic fibrosis. Several genes within this cluster are associated with hepatic fibrosis or HSC activation (Figure 3) [18].

Genes within clusters 2 and 4 also exhibited functional association with leukocyte extravasation signalling. Several genes with chemotactic activity for eosinophils (*CCL11*), neutrophils (*S100A8*, *S100A9* [19]) and monocytes/macrophages (*CCL6*, *CCL8*, *CCL12*, *CXCL14*) occur within these clusters and correlated well with the expression of genes encoding markers for eosinophils, neutrophils and alternatively activated macrophage markers, respectively (Table 2). Most striking was the marked up-regulation of *S100A8* and *S100A9* compared with uninfected controls at 6 (*S100A8*: 91-fold, p≤0.01; *S100A9*: 62-fold, p≤0.01) and 7 weeks p.i. (138-fold, p≤0.01; *S100A9*: 153-fold, p≤0.01). *CCL21a,b&c*, also occurred within this cluster and showed significant up-regulation compared with liver tissue from uninfected mice (4-fold, p≤0.01) only at 7 weeks p.i.

### Real-time PCR

The expression patterns established by real time PCR correlated well with the microarray data (Spearman's correlation; r = 0.74; p≤0.001) (Figure S1). Real-time PCR indicated that expression of *IL-4* (NM_021283) peaked at 6 weeks p.i. (24-fold relative to uninfected controls, p≤0.01) and declined thereafter. *IL-13* (NM_008355) expression peaked at 6 (5-fold, p≤0.001) and 7 weeks p.i. (4-fold, p≤0.001) during the development of fibrosis. *IL-10* (NM_010548) expression reached 5-fold (p≤0.01) at 6 weeks and 7-fold (p≤0.01) at 7 weeks p.i.

### Flow Cytometry

Adult worms numbers and hepatic egg burdens were identical in the separate time courses performed for microarray and flow cytometry analyses (t-test, p>0.05) (Figure S2).

**Figure 4:**
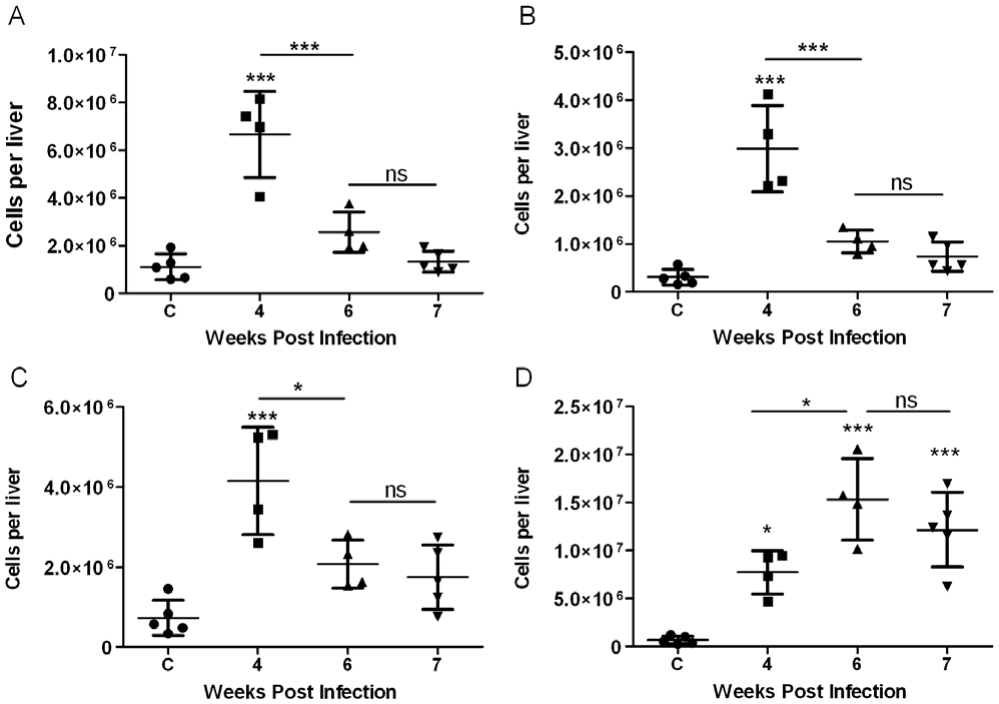
Temporal changes in the cellular composition of the *S. japonicum*-infected murine liver revealed by flow cytometry. Flow cytometry showed an increase in the number of CD4+ T-cells (CD4+CD3+) (A), CD8+ T-cells (CD8+CD3+) (B), and B-cells (CD19+) (C) in the liver at 4 weeks p.i, but there was a decline in the numbers of these cells thereafter. The number of macrophages in the liver (D) was significantly increased at 4 weeks p.i. compared with tissue from uninfected mice and the number of these cells continued to rise over time peaking at 6-7 weeks p.i. Values represent means ± 1SD. One mouse from the 4 and 6 weeks groups harboured no adult worms (i.e. non-infected) and was excluded from these analyses (n = 4). n = 5 all other groups. * p≤0.05, ** p≤0.01, ***p≤0.001 compared with uninfected liver unless otherwise indicated.

Analysis of the cellular composition of the liver revealed a significant increase in CD4+ T-cells, CD8+ T-cells and B-cells at 4 weeks p.i, compared with uninfected mice where the numbers of these cells in the liver declined thereafter (Figure 4a–c). The number of macrophages in the liver was significantly increased from 4 weeks p.i. compared with uninfected controls and peaked at 6–7 weeks p.i (Figure 4d).

### Immunohistochemistry

**Figure 5:**
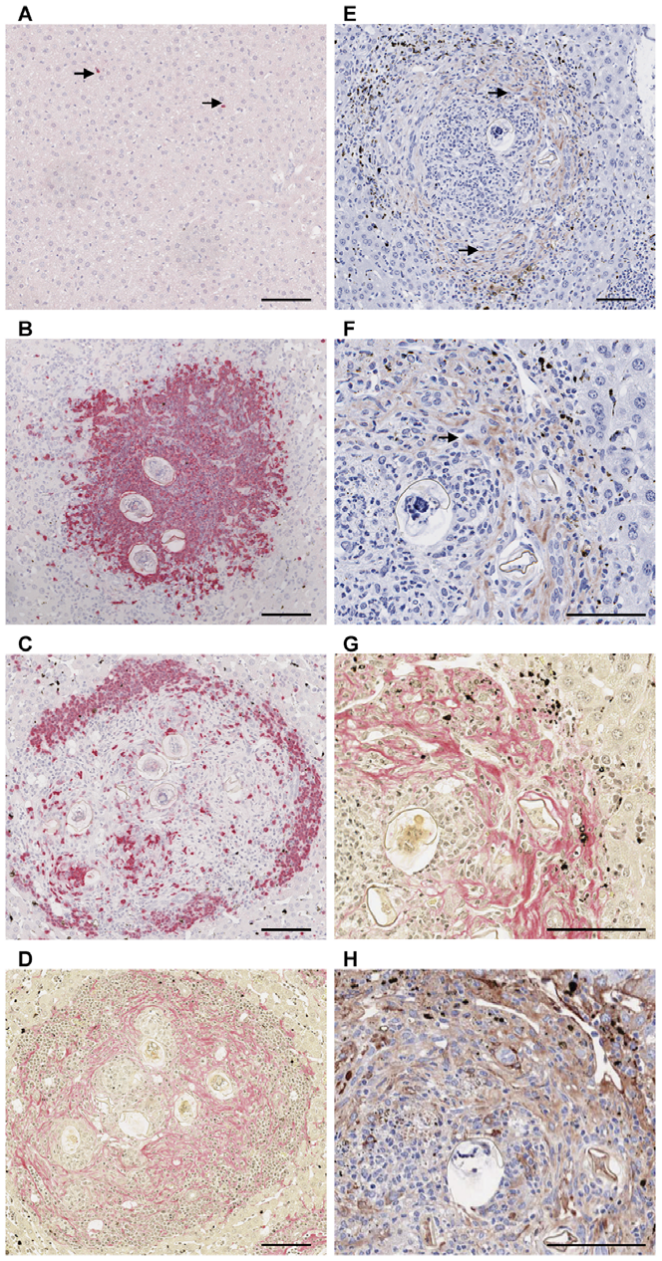
S100A8 and CCL7 localise to areas of known neutrophil accumulation and the fibrotic zone of *S. japonicum* induced granulomas, respectively. S100A8 positive (pink) cells occurred sporadically in the livers of uninfected mice (A; arrows; ×100), in the central region of established granulomas at 6 weeks p.i. (B; ×100) and at the periphery of granulomas (C; ×100) adjacent to fibrotic areas identified by Pico Sirius Red staining for collagen (pink) (D; ×100) at 7 weeks p.i. Whereas, uninfected liver was negative, CCL7 (Red-brown/arrows) was significantly increased at 7 weeks p.i. and localised to areas of fibrosis (E; ×100, & F; ×200), as identified by Pico Sirius Red staining for collagen (G; ×200), resembling the distribution of α-SMA staining (H; ×200). Scale bar equals 100 µm.

Due to the striking up-regulation of S100A8 and the previous association of CCL7 with fibrosis [20], immunohistochemistry was used to investigate the distribution of these two chemokines within granulomatous liver tissue. Positive staining for S100A8 was remarkably similar to that for neutrophils. S100A8 positive cells occurred sporadically in the uninfected liver (Figure 5a). Focal clusters of positively stained cells were observed in the liver at 4 weeks p.i. and staining intensity increased significantly thereafter. At 6 and 7 weeks p.i, S100A8 positive cells localised to the inner most zone of established granulomas, where inflammatory cells including neutrophils and macrophages are known to accumulate [4] (Figure 5b), and to the periphery of more mature granulomas adjacent to the fibrotic areas (Figure 5c) as confirmed by staining for collagen (Figure 5d). CCL7 was absent from uninfected livers but was present in infected livers from 6 weeks p.i and was significantly elevated above control levels at 7 weeks p.i. Staining occurred predominantly in granulomas at the periphery of the liver (Figure 5e,f) and localised to the fibrotic zone of these lesions (Figure 5g) resembling the distribution of HSCs (Figure 5h).

## Discussion

We used whole genome microarray analysis and real-time PCR combined with histology and flow cytometry to build a more integrated and global view of the gene signalling pathways and pathological mechanisms induced during *S. japonicum* infection than has been described previously. Our analyses confirm the development of a Th2 response during *S. japonicum*-induced granuloma formation and fibrosis, characterised by up-regulation of Th2 associated genes. Notably, we also observed sustained up-regulation of Th1 associated genes including the Th1 cytokine IFN- γ during granuloma development as previously reported in *S. japonicum* infection [21]. Together this suggests that the localised immune response to *S. japonicum* eggs in the liver may be of a mixed Th1/Th2 phenotype. Further, the early up-regulation of Th1 associated chemokines [22],[23] in our study suggests that this arm of the response may be important in the early recruitment of inflammatory cells to the liver and the initiation of the granulomatous response.

**Figure 6:**
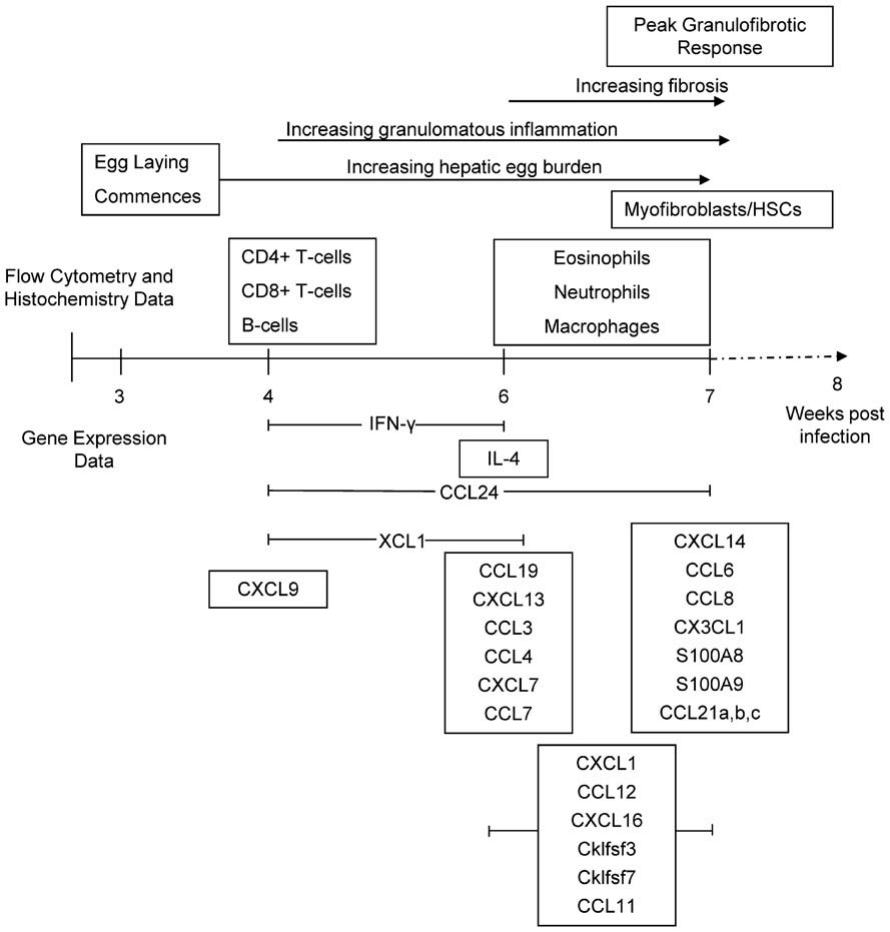
Summary of chemokines with distinct functions that exhibited peak expression during different phases of the *S. japonicum*-induced granulofibrotic response. Chemokines up-regulated earlier were predominantly T- and B-cell chemoattractants. Chemokines up-regulated in the mid-late stages of infection are associated with HSC recruitment and activation. Chemokines with peak expression late in infection were predominantly macrophage and neutrophil chemoattractants. The activity of these chemokines was reflected in the temporal recruitment of T-cells, B-cells, eosinophils, neutrophils, macrophages and myofibroblasts/HSCs to the granulomatous liver.

Widespread down-regulation of multiple components of many metabolic pathways in the livers of *S. japonicum* infected mice is indicative of a generalised down-regulation of the metabolic functions of the liver. These observations are consistent with those from *S. mansoni* infections and likely reflects increasing impairment of liver function associated with progressive tissue damage [24]. Genes up-regulated during infection were temporally associated with distinct biological functions. This was especially true for chemokines with activity for distinct cell types which were up-regulated during different phases of the granulofibrotic response (Figure 6).

Up-regulated gene expression in the early phase of *S. japonicum*-induced granuloma formation (4–6 weeks p.i) was primarily associated with antigen presentation and cell recruitment. Chemokines up-regulated during this phase were predominantly T-cell and B-cell chemoattractants [25],[26],[27],[28],[29] and their activity was reflected by a concurrent increase in the expression of T- and B-cell makers and the early recruitment of CD4+ T-cells, CD8+ T-cells and B-cells into the liver, consistent with previous studies showing that these cells are required for the development of *S. japonicum* induced granulomas [30],[31]. Peak expression of the B-cell chemokine *CXCL13* correlated well with the expression of the B-cell maturation marker *CD22* but occurred after the influx of CD19+ B-cells into the liver. These results suggest that CXCL13 may not be crucial for the recruitment of B-cells to the liver but may be associated with maturation and/or retention of activated B-cells in the liver. Similar roles for CXCL9, CXCL13 and CXCL16 in T- and B-cell recruitment have been suggested in other models of liver disease including chronic Hepatitis C, Hepatocellular Carcinoma, Primary Biliary Cirrhosis and Primary Sclerosing Choliangitis [32],[33],[34]. We also observed early and sustained up-regulation of the eosinophil chemoattractant *CCL24*, which is likely contributing to the ongoing recruitment of eosinophils to the liver as illustrated histologically (Figure 2). The related chemokine *CCL11* was significantly up-regulated later in infection mirroring the accumulation of eosinophils in the liver and correlating well with the expression of eosinophil-associated genes. The late up-regulation of *CCL11* suggests a role in the recruitment of eosinophils during the fibrotic response, an observation consistent with the association of this chemokine with fibrosis in chronic liver disease [35].

In accordance with its proposed role in promoting *S. mansoni*-induced granuloma formation [2], *CCL3* expression peaked during granuloma development. Further, we show a correlation between the expression of *CCL3*, several procollagen genes and the profibrotic cytokines *TGF-β1* and *PDGF- β*. This is in agreement with a recent study implicating CCL3 in the development of bleomycin-induced pulmonary fibrosis in the mouse where CCL3 was shown to regulate the recruitment of TGF-β1-producing macrophages and bone-marrow-derived fibrocytes [36]. Together, these results suggest that CCL3 may promote *S. japonicum*-induced fibrogenesis via a mechanism similar to that proposed for bleomycin-induced pulmonary fibrosis [36]. The related chemokine *CCL4* had a similar expression pattern suggesting that it may also play a role in fibrogenesis.

Toll-like receptor (TLR) signalling was a prominent feature of the mid-late phase of the granulofibrotic response. TLR4 signalling has been shown to be critical to the development of hepatic fibrosis in several experimental models [37]. Further, signalling through TLR4 on quiescent HSCs sensitises these cells to TGF-β1 thereby inducing their activation, chemokine secretion and the chemotaxis of Kupffer cells [38]. The co-incidence of peak *TLR* expression with that of the pro-fibrogenic cytokines *IL-13* and *TGF-β1* in our study implies that TLR signalling may be involved in the development of *S. japonicum*-induced fibrosis.

Expression of the chemokines *CCL21*, *CXCL1*, *CCL7* and *CCL12* showed a significant correlation with the expression of procollagen genes and resembled the recruitment of HSCs into granulomas. These chemokines have been shown to be chemoactive for HSCs or myofibroblasts and have been implicated in the pathogenesis of a variety of fibrotic diseases [39],[40],[41],[42]. CCL21, CCL7 and CXCL1 are also known to directly induce the activation and wound healing responses of HSCs or myofibroblasts [20],[39],[40]. As well, CCL7 was recently shown to work synergistically with TGF-β1 to induce collagen production in dermal fibroblasts [43]. Furthermore, we showed that CCL7 expression correlates with the expression of fibrosis associated genes and localises to the fibrotic zone of granulomas with a similar distribution to HSCs during *S. japonicum* infection (Figure 5e–h). The induction of *CCL7* and *CCL12* during fibrosis is consistent with the role of the related chemokine CCL2 in other liver diseases [44]. CCL2 is a chemoattractant for HSCs [44] and its expression is associated with fibrogenesis in both human cholestatic liver disease and in the bile-duct-ligated rat model of cholestatic liver injury [45]. CCL2 expression is important for the development of granulomas and fibrosis in schistosomiasis mansoni [46]. In contrast, there was no significant change in its expression during *S. japonicum* infection; instead, CCL21, CXCL1, CCL12 and particularly CCL7, may promote the initiation of *S. japonicum* induced fibrosis by recruiting fibrogenic effector cells and by directly inducing fibrogenic signalling pathways.

Genes with peak expression later in the granulofibrotic response (7 weeks p.i) included many genes associated with fibrosis, such as matrix metalloproteinases (MMPs) and tissue inhibitors of matrix metalloproteinases (TIMPs). MMPs and TIMPs play an important role in remodelling of fibrotic tissue and the ratio of MMP:TIMP expression may be a determining factor in the outcome and severity of schistosome induced fibrosis [47]. Up-regulation of *MMP-2*, *MMP-9*, *MMP-13*, *TIMP-1* and *TIMP-2* appears to be common to both *S. japonicum* and *S. mansoni* induced fibrosis [13],[47]. In contrast, we observed no change in the expression of *MMP-8* or *MMP-12*, although expression of these genes has been shown to correlate with peak fibrosis during *S. mansoni* infection [13],[47]. This is the first report of the up-regulation of *MMP-23* and *MMP-25* during murine schistosomiasis. These dissimilarities in MMP expression are suggestive of differences in the wound healing response during *S. mansoni* and *S. japonicum* infection, which may partly explain the differing degree of fibrosis induced by these two parasites.

Chemokines whose peak expression correlated with peak fibrosis and the expression of *COL1A1* were predominantly macrophage and neutrophil chemoattractants. *CXCL14*, a chemokine which in humans is chemoactive for monocytes [48], showed peak expression of 10-fold at 7 weeks and therefore may also contribute to the recruitment of monocytes/macrophages to granulomas. This is the first report of the up-regulation of *CXCL14* during schistosome infection. Expression of macrophage chemokines corresponded with the up-regulation of macrophage genes and alternatively activated macrophage markers and coincided with the accumulation of F4/80+ macrophages in the liver. Together these results indicate these chemokines contribute to the recruitment of macrophages to the liver during *S. japonicum* infection and that these macrophages are of an alternatively activated phenotype in accord with the proposed role of these cells in regulating *S. mansoni* induced inflammation and fibrosis, and other Th2 dominant inflammatory diseases [49],[50]. Up-regulation of *CCL6*, *CCL7* and *CCL8* has previously been reported for *S. mansoni* infection [10],[13],[22] and so might represent a common mechanism whereby macrophages are recruited into schistosome-induced granulomas.

The marked up-regulation of neutrophil chemokines and the number of neutrophils in the liver at 7 weeks p.i. was striking. Neutrophil recruitment has been associated with the development of fibrosis in a number of other chronic liver diseases [51],[52]. The precise role of these cells in the fibrotic response, however, remains ambiguous. Harty *et al* [51] reported a role for macrophage mediated neutrophil recruitment in the resolution of fibrosis in a rat model of cholestatic liver disease. In contrast, other studies, including Th1 and Th2 polarised models of *S. mansoni* infection, have reported an association between increased neutrophil accumulation and the up-regulated expression of *CXCL1* with the development of severe disease [52],[53],[54]. We localised S100A8 and neutrophils to an area adjacent to the fibrotic zone of mature granulomas (Figure 5b,c), which suggests an accessory role for these cells in *S. japonicum* induced fibrosis. Whether this role is in promoting or regulating the fibrotic response remains to be determined.

In summary, we present the most comprehensive study to date of the transcriptional profile of the schistosome infected liver in the mouse model. It shows that cellular recruitment to granulomatous tissue is tightly regulated by the temporal expression of distinct chemokine subsets and details for the first time the up-regulation of *CXCL7* and *CXCL14* during schistosome-induced granuloma formation. Additionally, further evidence is provided that there are discrete differences in the cytokine, chemokine and wound healing responses in *S. japonicum* and *S. mansoni* infections and indicates that neutrophils may play a significant role in determining the outcome of *S. japonicum* induced pathology. Furthermore, similarities between the results presented here for schistosomiasis japonica and other chronic inflammatory diseases of the liver suggest that common immunopathogenic pathways regulate the development of hepatic fibrosis in a variety of pathologies.

## Supporting Information

Figure S1 Real-time PCR confirms expression profiles obtained by microarray analysis. Expression of a subset of genes analysed by real-time PCR is depicted in the line graphs and is displayed as fold change relative to uninfected liver (C) at 4, 6 and 7 weeks p.i. Colour bars are representative of corresponding microarray data where down-regulation is coloured green, up-regulated expression is coloured red and unchanged expression is coloured black. * = p≤0.05, ** = p≤0.01, ***p≤0.001 in comparison to uninfected liver.
(1.46 MB TIF)

Figure S2 Parasite burdens for microarray and flow cytometry were identical. A: Infected mice harboured a mean of 5 adult worm pairs. Eggs were first seen in the liver at four weeks p.i and hepatic egg burden increased significantly there after. B: There was no difference in the adult worm burden or hepatic egg burden in mice used in the separate time courses performed for microarray analysis (Grey) and flow cytometry (White) (t-test, p>0.05). Values represent mean values from 4 mice used for flow cytometry or for microarray analysis ±1SD *p≤0.05, **p≤0.01, ***p≤0.001, ns = not significant. Legend: M = microarray; F = flow cytometry.
(2.05 MB TIF)

Table S1 Real time PCR primers used in the study. Primers were designed using Primer3 software or sourced from the literature 1. Chiu B-C, et al. Am. J. Respir. Cell Mol. Biol. 2003;29:106–116. 2. Pelosof LC, et al. Cell Microbiol 2006;8:508–522. 3. Rodriguez A, et al. BMC Genomics 2007;8:379. 4. Sandler NG, et al. J Immunol 2003;171:3655–3667. 5. Hesse M, et al. J Immunol 2004;172:3157–3166. 6. Amante F et al Am J Pathol 2007;171:548–559.
(0.05 MB DOC)

Table S2 Temporal clustering of gene expression is associated with distinct biological functions. *Top 5 Biological functions/Disorders for each hierarchical cluster as identified by Ingenuity Pathway Analysis. P-values represent the range of p-values for lower level functions within these categories.
(0.06 MB DOC)

Table S3 *S. japonicum* infection is associated with the temporal expression of genes involved in distinct biological signalling pathways. *Top 5 Canonical Pathways for each hierarchical cluster as identified by Ingenuity Pathway Analysis. (n/a: no genes within this cluster that met the specified filtering criteria were associated with this pathway at this time point).
(0.07 MB DOC)
