# Supplementary figures and images for: Temporal Expression of Chemokines Dictates the Hepatic Inflammatory Infiltrate in a Murine Model of Schistosomiasis
Source: PLoS Negl Trop Dis. 2010 Feb 9;4(2):e598. doi: 10.1371/journal.pntd.0000598 (PMC2817718; doi:10.1371/journal.pntd.0000598)

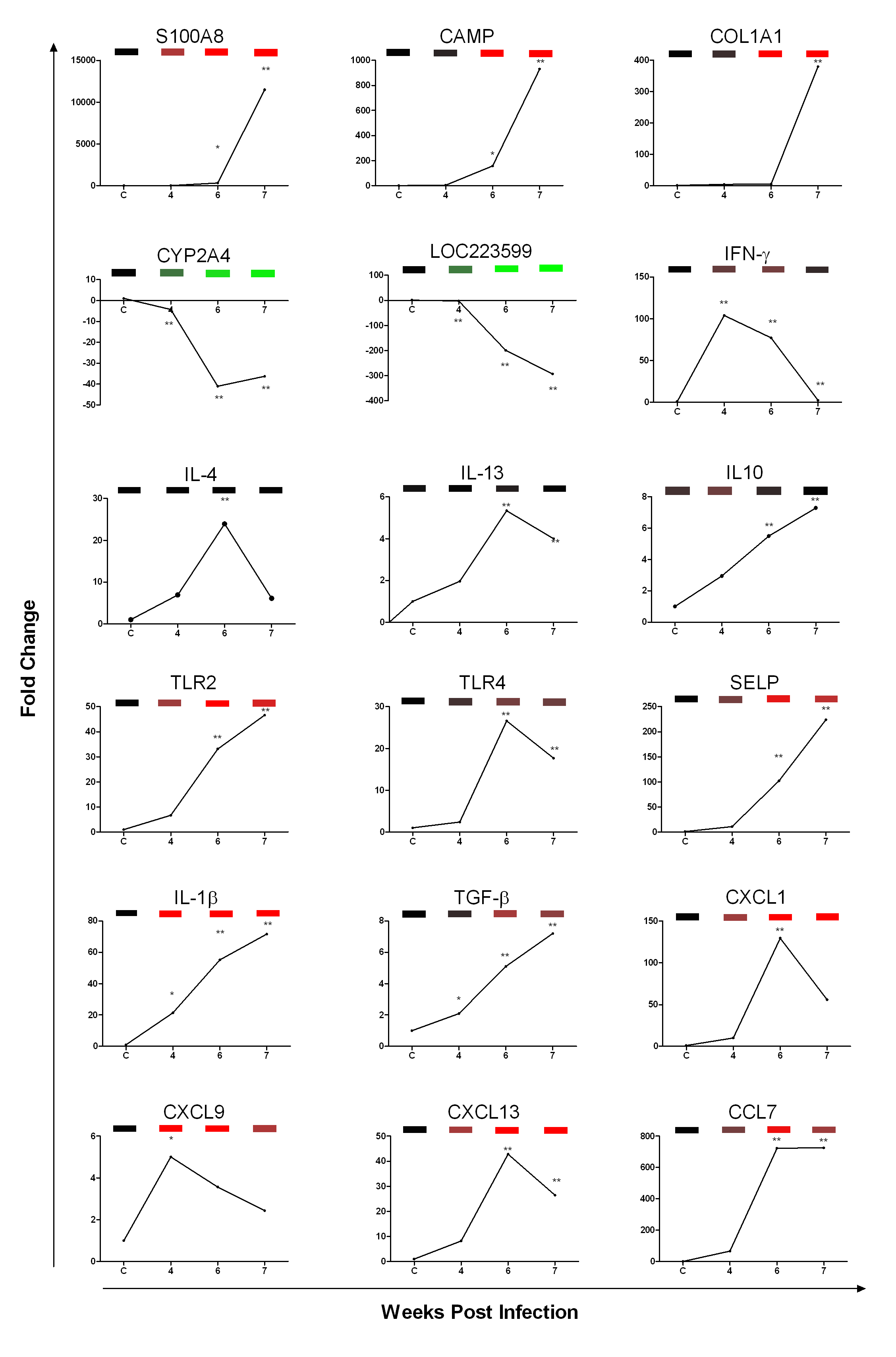

Supplement: Figure S1 — Real-time PCR confirms expression profiles obtained by microarray analysis. Expression of a subset of genes analysed by real-time PCR is depicted in the line graphs and is displayed as fold change relative to uninfected liver (C) at 4, 6 and 7 weeks p.i. Colour bars are representative of corresponding microarray data where down-regulation is coloured green, up-regulated expression is coloured red and unchanged expression is coloured black. * = p≤0.05, ** = p≤0.01, ***p≤0.001 in comparison to uninfected liver. (1.46 MB TIF) [file pntd.0000598.s001.tif]

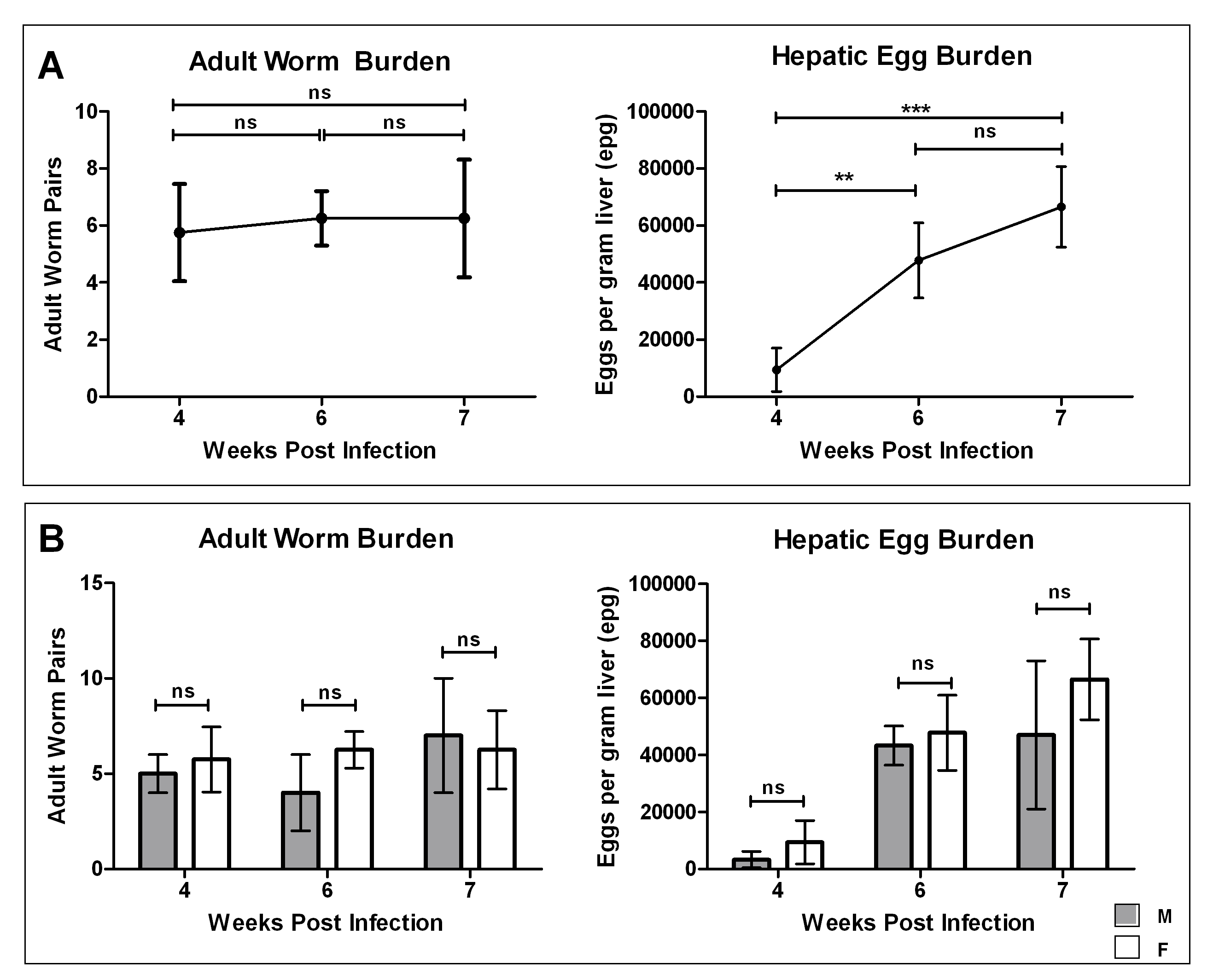

Supplement: Figure S2 — Parasite burdens for microarray and flow cytometry were identical. A: Infected mice harboured a mean of 5 adult worm pairs. Eggs were first seen in the liver at four weeks p.i and hepatic egg burden increased significantly there after. B: There was no difference in the adult worm burden or hepatic egg burden in mice used in the separate time courses performed for microarray analysis (Grey) and flow cytometry (White) (t-test, p>0.05). Values represent mean values from 4 mice used for flow cytometry or for microarray analysis ±1SD *p≤0.05, **p≤0.01, ***p≤0.001, ns = not significant. Legend: M = microarray; F = flow cytometry. (2.05 MB TIF) [file pntd.0000598.s002.tif]
