# Supplementary material for: Temporal Expression of Chemokines Dictates the Hepatic Inflammatory Infiltrate in a Murine Model of Schistosomiasis
Source: PLoS Negl Trop Dis. 2010 Feb 9;4(2):e598. doi: 10.1371/journal.pntd.0000598 (PMC2817718; doi:10.1371/journal.pntd.0000598)
Supplement: Table S1 — Real time PCR primers used in the study. Primers were designed using Primer3 software or sourced from the literature 1. Chiu B-C, et al. Am. J. Respir. Cell Mol. Biol. 2003;29:106–116. 2. Pelosof LC, et al. Cell Microbiol 2006;8:508–522. 3. Rodriguez A, et al. BMC Genomics 2007;8:379. 4. Sandler NG, et al. J Immunol 2003;171:3655–3667. 5. Hesse M, et al. J Immunol 2004;172:3157–3166. 6. Amante F et al Am J Pathol 2007;171:548–559. (0.05 MB DOC) [file pntd.0000598.s003.doc]

| **Gene** | **Forward Primer** | **Reverse Primer** | **Amplicon Length** | **Source*** |
| --- | --- | --- | --- | --- |
| TLR4 | 5’ ggc agc agg tgg aat tgt at | 5’ agg ccc cag agt ttt gtt ct | 198 | Primer3 |
| CXCL9 | 5’ ctg agg ctc acg tca cca atc | 5’ ggc tct agg ctg acc caa atg | 110 | 1 |
| TGFβ | 5’ tgc gct tgc aga gat taa aa | 5’ gct gaa tcg aaa gcc ctg ta | 197 | Primer3 |
| CXCL1 | 5’ gct ggg att cac ctc aag aa | 5’ tct ccg tta ctt ggg gac ac | 180 | Primer3 |
| CXCL13 | 5’ tac cca acc cac atc ctt gt | 5’ cca tct cgc aaa cct ctt gt | 190 | Primer3 |
| CCL7 | 5’ gtg tcc ctg gga agc tgt ta | 5’ aga aag aac agc ggt gag ga | 195 | Primer3 |
| TLR2 | 5’ gga act gtc gga ggt aga gtt cg | 5’ ttt cta ctt tac cca gct cgc tca | 101 | 2 |
| SELP | 5’ agc agg gac act gac aat cc | 5’ ttg ggt cat atg cag cgt ta | 200 | Primer3 |
| IL1β | 5’ gag tgt gga tcc caa gca at | 5’ acg gat tcc atg gtg aag tc | 201 | Primer3 |
| CYP2A4 | 5’ gac cga atg aag atg ccc ta | 5’ tga agt ctt tgg ggt tgg ag | 202 | Primer3 |
| LOC22359 | 5’ gga ccg aag cag tct ttg ag | 5’ ggc ttc ctg acc cag atg ta | 197 | Primer3 |
| CSAD | 5’ ctg cgt agt ctc ctg ggt tc | 5’ ccc cca atg ctg gta gta aa | 199 | Primer3 |
| HBB-1 | 5’ tga gct cca ctg tga caa gc | 5’ ttg ttc aca ggc aag agc ag | 205 | Primer3 |
| S100A8 | 5’ gga aat cac cat gcc ctc tac | 5’ gcc aca ccc act ttt atc acc | 173 | 3 |
| CAMP | 5’ gtc ttg gga acc atg cag tt | 5’ cag gtc cag gag acg gta ga | 186 | Primer3 |
| COL1A1 | 5’ aac tgg act gtc cca acc cc | 5’ tcc ctc gac tcc tac atc ttc tg | 100 | 4 |
| IL4 | 5’ acg agg tca cag gag aag gga | 5’ agc cct aca gac gag ctc act c | 101 | 5 |
| IL13 | 5’ ggc agc atg gta tgg agt gtg | 5’ tgg gtc ctg tag atg gca ttg | 101 | 5 |
| IL10 | 5’ ata act gca ccc act tcc cag tc | 5’ ccc aag taa ccc tta aag tcc tgc | 154 | 5 |
| IL5 | 5’ tga caa gca at gaga cga tga gg | 5’ acc ccc acg gac agt ttg att c | 116 | 5 |
| HPRT | 5’gtt gga tac agg cca gac ttt gtt g | 5’ gat tca act tgc gct cat ctt agg c | 163 | 6 |
