# Supplementary material for: Temporal Expression of Chemokines Dictates the Hepatic Inflammatory Infiltrate in a Murine Model of Schistosomiasis
Source: PLoS Negl Trop Dis. 2010 Feb 9;4(2):e598. doi: 10.1371/journal.pntd.0000598 (PMC2817718; doi:10.1371/journal.pntd.0000598)
Supplement: Table S2 — Temporal clustering of gene expression is associated with distinct biological functions. *Top 5 Biological functions/Disorders for each hierarchical cluster as identified by Ingenuity Pathway Analysis. P-values represent the range of p-values for lower level functions within these categories. (0.06 MB DOC) [file pntd.0000598.s004.doc]

| **Cluster** | **Top Higher level Biological Functions/ Disorders*** | **P value** |
| --- | --- | --- |
| 1 | 1. Lipid Metabolism | 4.53x10^-5^ - 4.35x10^-2^ |
|  | 2. Small Molecule Biochemistry | 4.53x10^-5^ - 4.89x10^-2^ |
|  | 3. Cell Morphology | 7.53x10^-5^ - 4.89x10^-2^ |
|  | 4. Connective Tissue Development and Function | 7.53x10^-5^ - 4.35x10^-2^ |
|  | 5. Cell Signalling | 2.69x10^-4^ - 3.18x10^-2^ |
| 2 | 1. Immunological Disease | 2.16x10^-20^ - 9.28x10^-4^ |
|  | 2. Inflammatory Disease | 1.30x10^-19^- 8.94x10^-4^ |
|  | 3. Connective Tissue Disorders | 4.31x10^-19^ - 9.28x10^-4^ |
|  | 4. Skeletal and Muscular Disorders | 3.36x10^-18^ - 9.28x10^-4^ |
|  | 5. Cellular Movement | 1.68x10^-16^ - 8.94x10^-4^ |
| 3 | 1. Immunological Disease | 1.15x10^-21^ - 4.42x10^-4^ |
|  | 2. Cellular Movement | 2.31x10^-19^ - 5.67x10^-4^ |
|  | 3. Haematological System Development and Function | 2.31x10^-19^ - 5.67x10^-4^ |
|  | 4. Immune Response | 2.31x10^-19^ - 5.67x10^-4^ |
|  | 5. Immune and Lymphatic System Development and Function | 2.04x10^-17^ - 5.05x10^-4^ |
| 4 | 1. Cancer | 1.04x10^-13^ - 9.36x10^-3^ |
|  | 2. Cellular Movement | 2.1x10^-11^- 9.36x10^-3^ |
|  | 3. Cell-to-Cell Signalling and Interaction | 5.44x10^-11^ - 8.16x10^-3^ |
|  | 4. Reproductive System Disease | 1.84x10^-9^ - 8.87x10^-3^ |
|  | 5. Skeletal and Muscular Disorders | 4.34x10^-9^ - 8.70x10^-3^ |
| 5 | 1. Immunological Disease | 9.29x10^-31^ - 9.78x10^-4^ |
|  | 2. Connective Tissue Disorders | 1.01x10^-23^ - 8.92x^10-4^ |
|  | 3. Inflammatory Disease | 1.01x10^-23^ - 8.92x^10-4^ |
|  | 4. Skeletal and Muscular Disorders | 1.01x10^-23^ - 8.92x10^-4^ |
|  | 5. Cellular Growth and Proliferation | 4.37x10^-21^ - 9.03x10^-4^ |
| 6 | 1. Lipid Metabolism | 3.03x10^-11^ - 1.85x10^-2^ |
|  | 2. Small Molecule Biochemistry | 3.03x10^-11^ - 1.85x10^-2^ |
|  | 3. Metabolic Disease | 1.12x10^-9^ - 1.85x10^-2^ |
|  | 4. Amino Acid Metabolism | 1.01x10^-7^ - 1.85x10^-2^ |
|  | 5. Molecular Transport | 1.24x10^-6^ - 1.85x10^-2^ |
| 7 | 1. Post-translational Modification | 9.43x10^-4^ - 1.59x10^-2^ |
|  | 2. Protein Folding | 9.43x10^-4^ - 3.77x10^-3^ |
|  | 3. Drug Metabolism | 1.89x10^-3^ - 4.71x10^-3^ |
|  | 4. Gene Expression | 1.89x10^-3^ - 2.83x10^-3^ |
|  | 5. Amino Acid Metabolism | 2.83x10^-3^ - 2.15x10^-2^ |
