# Supplementary material for: Temporal Expression of Chemokines Dictates the Hepatic Inflammatory Infiltrate in a Murine Model of Schistosomiasis
Source: PLoS Negl Trop Dis. 2010 Feb 9;4(2):e598. doi: 10.1371/journal.pntd.0000598 (PMC2817718; doi:10.1371/journal.pntd.0000598)
Supplement: Table S3 — S. japonicum infection is associated with the temporal expression of genes involved in distinct biological signalling pathways. *Top 5 Canonical Pathways for each hierarchical cluster as identified by Ingenuity Pathway Analysis. (n/a: no genes within this cluster that met the specified filtering criteria were associated with this pathway at this time point). (0.07 MB DOC) [file pntd.0000598.s005.doc]

| **Cluster** | **Top Canonical Pathways*** |  | **P-value** |  |
| --- | --- | --- | --- | --- |
|  |  | **4 wks** | **6 wks** | **7 wks** |
| 1 | 1. Clathrin-mediated Endocytosis | 7.58x10^-4^ | n/a | n/a |
|  | 2. Toll-like Receptor Signalling | 7.56x10^-3^ | n/a | n/a |
|  | 3. Nitric Oxide Signalling in the Cardiovascular System | 1.45x10^-2^ | n/a | n/a |
|  | 4. p53 Signalling | 2.04x10^-2^ | n/a | n/a |
|  | 5. Nicotinate and Nicotinamide Metabolism | 2.49x10^-2^ | n/a | n/a |
| 2 | 1. Leukocyte Extravasation Signalling | 2.55x10^-1^ | 4.22x10^-6^ | 4.98x10^-6^ |
|  | 2. Actin Cytoskeleton Signalling | n/a | 3.93x10^-3^ | 1.45x10^-4^ |
|  | 3. Fcγ Receptor-mediated Phagocytosis in Macrophages and Monocytes | n/a | 6.72x10^-4^ | 1.96x10^-4^ |
|  | 4. Hepatic Fibrosis/ Hepatic Stellate Cell Activation | n/a | 3.23x10^-3^ | 2.54x10^-4^ |
|  | 5. Sonic Hedgehog Signalling | n/a | 7.8x10^-4^ | 1.06x10^-3^ |
| 3 | 1. Leukocyte Extravasation Signalling | 2.68x10^-10^ | 2.48x10^-11^ | 5.53x10^-12^ |
|  | 2. Natural Killer Cell Signalling | 2.83x10^-6^ | 3.02x10^-6^ | 1.44x10^-6^ |
|  | 3. Fc Epsilon RI Signalling | 3.84x10^-6^ | 3.24x10^-5^ | 1.69x10^-5^ |
|  | 4. Fcγ Receptor-mediated Phagocytosis in Macrophages and Monocytes | 3.84x10^-6^ | 6.69x10^-9^ | 2.69x10^-8^ |
|  | 5. B-cell Receptor Signalling | 6.26x10^-6^ | 1.5x10^-7^ | 5.39x10^-8^ |
| 4 | 1. Hepatic Fibrosis/ Hepatic Stellate Cell Activation | n/a | 5.85x10^-3^ | 6.57x10^-3^ |
|  | 2. Leukocyte Extravasation Signalling | n/a | 8.4x10^-2^ | 3.43x10^-3^ |
|  | 3. Glutathione Metabolism | n/a | 4.57x10^-3^ | 3.64x10^-3^ |
|  | 4. Eicosanoid Signalling | n/a | 5.01x10^-2^ | 4.69x10^-3^ |
|  | 5. IL-8 Signalling | n/a | 7.29x10^-2^ | 1.02x10^-2^ |
| 5 | 1. Antigen Presentation Pathway | 3.04x10^-15^ | 8.32x10^-15^ | 1.33x10^-11^ |
|  | 2. Interferon Signalling | 1.93x10^-7^ | 2.85x10^-8^ | 2.17x10^-4^ |
|  | 3. T-cell Receptor Signalling | 8.72x10^-6^ | 5.85x10^-3^ | 6.69x10^-5^ |
|  | 4. IL-4 Signalling | 1.58x10^-5^ | 5.84x10^-6^ | 8.55x10^-6^ |
|  | 5. Hepatic Fibrosis/ Hepatic Stellate Cell Activation | 4.09x10^-5^ | 2.43x10^-8^ | 2.40x10^-4^ |
| 6 | 1. Fatty Acid Metabolism | 8.04x10^-6^ | 8.71x10^-28^ | 2.13x10^-31^ |
|  | 2. Metabolism of Xenobiotics by Cytochrome P450 | 7.84x10^-5^ | 2.71x10^-23^ | 6.67x10^-29^ |
|  | 3. LPS/IL-1 Mediated Inhibition of RXR Function | 6.4x10^-14^ | 6.12x10^-23^ | 1.03x10^-26^ |
|  | 4. Tryptophan Metabolism | 5.52x10^-5^ | 5.09x10^-24^ | 1.93x10^-25^ |
|  | 5. Valine, Leucine and Isoleucine Degradation | 6.16x10^-3^ | 3.03x10^-18^ | 1.15x10^-19^ |
| 7 | 1. Protein Ubiquitination Pathway | 5.32x10^-4^ | n/a | n/a |
|  | 2. Methionine Metabolism | 2.06x10^-2^ | n/a | n/a |
|  | 3. Interferon Signalling | 2.33x10^-2^ | n/a | n/a |
|  | 4. Role of RIG1-like Receptors in Antiviral Innate Immunity | 3.16x10^-2^ | 3.56x10^-3^ | n/a |
|  | 5. Activation of IRF by Cytosolic Pattern Recognition Receptors | 4.89x10^-2^ | 5.55x10^-3^ | n/a |
